# *Citrus sinensis* Essential Oils an Innovative Antioxidant and Antipathogenic Dual Strategy in Food Preservation against Spoliage Bacteria

**DOI:** 10.3390/antiox12020246

**Published:** 2023-01-21

**Authors:** Marilina Manzur, María C. Luciardi, M. Amparo Blázquez, María R. Alberto, Elena Cartagena, Mario E. Arena

**Affiliations:** 1 INBIOFAL (Instituto de Biotecnología Farmacéutica y Alimentaria) CONICET, Avenida Kirchner 1900, Tucumán 4000, Argentina; 2 Facultad de Bioquímica, Química y Farmacia, Universidad Nacional de Tucumán (UNT), Ayacucho 471, Tucumán 4000, Argentina; 3 Departament de Farmacologia, Facultat de Farmàcia, Universitat de València, Avd. Vicent Andrés Estellés s/n, 46100 Burjasot, Spain

**Keywords:** sweet orange, cold pressing, hydrodistillation, scavenging activity, reducing capacity, virulence factors, quorum sensing

## Abstract

The present study evaluates the chemical compositions and antioxidant and antipathogenic properties of commercial orange (*Citrus sinensis* (L.) Osbeck) essential oils obtained using the cold-press method (EOP) and the cold-press method followed by steam distillation (EOPD). The chemical compositions of the volatilizable fractions, determined by gas chromatography-mass spectrometry, were similar in both samples. A relatively large amount of *γ*-terpinene was found in the EOPD (1.75%) as compared to the EOP (0.84%). Monoterpene hydrocarbons with limonene (90.4–89.8%) followed by myrcene (3.2–3.1%) as the main compounds comprised the principal phytochemical group. The non-volatile phenolics were eight times higher in the EOP than in the EOPD. Several assays with different specificity levels were used to study the antioxidant activity. Although both essential oils presented similar reducing capacities, the radical elimination ability was higher for the EOP. Regarding the antipathogenic properties, the EOs inhibited the biomass and cell viability of *Staphylococcus aureus* and *Pseudomonas aeruginosa* biofilms. Furthermore, both EOs similarly attenuated the production of elastase, pyocyanin, and quorum-sensing autoinducers as assessed using Gram-negative bacteria. The EOP and EOPD showed important antioxidant and antipathogenic properties, so they could represent natural alternatives to extend the shelf life of food products by preventing oxidation and contamination caused by microbial spoilage.

## 1. Introduction

Different industries, such as the pharmaceutical, sanitary, cosmetic, and food industries, have paid attention to essential oils (EOs) to improve the shelf life and quality of products due to their potent antimicrobial and antioxidant activities [1,2]. As a result, the application of naturally produced antimicrobial compounds, such as EOs extracted from plants, has received significant attention [3,4,5,6]. EOs are mixtures of 20–100 different plant secondary metabolites with significant chemical variability [7]. This chemical variability is due to the variable ecological and geographical conditions, the age of the plant, the harvesting time, and the different extraction methodologies. The variations in the chemical profiles of EOs may influence their biological activity [8]. In numerous cases, the EOs’ bioactivities are attributed to one or two principal components. However, the major constituents sometimes do not represent the overall activity [8,9].

Several EOs, such as *Citrus* EOs, have obtained the “GRAS” (Generally Regarded as Safe) category from the US Food and Drug Administration, given their favorable safety profiles [8], which is why studies on the biological activities of *Citrus* essential oils are increasing. *Citrus* spp. have been extensively investigated for their EOs, although their biological activities are still under study [3]. *Citrus* species belonging to the Rutaceae family are among the most commercially significant crops cultivated in tropical and subtropical climate regions [10]. The orange is one of the top-rated citrus fruits, and orange production accounts for more than 50% of global citrus production [11]. *Citrus* essential oils are particularly fascinating since they can be used as antioxidants because of their ability to protect organisms and tissues from the damage inflicted by reactive oxygen species and as flavoring agents [12]. They are rich sources of bioactive compounds; about 85–99% of the components are volatile and include a mixture of monoterpenes, sesquiterpenes, and oxygenated derivatives (aldehydes, ketones, acids, alcohols, and esters) [13]. The EOs of citrus fruits of various species have shown various biological activities, such as antibacterial, antiviral, fungicidal, and antioxidant effects. Therefore, these EOs can be used as a safer alternative to synthetic preservatives [14,15].

Another critical problem in the food industry is the tolerance of foodborne pathogens to various environmental stressors used as preservation methods (heat, cold, salt, and acid conditions), as well as the pathogens’ ability to form biofilms on biotic or abiotic surfaces. The biofilm allows bacteria to contaminate surfaces in contact with food and transfer onto them [16]. Several foodborne disease outbreaks have been associated with biofilms [17], which has become a significant challenge to food production [18,19]. In biofilm formation, quorum sensing (QS) enables a phenotypic change in bacteria, whereby sessile biofilm bacteria show increased resistance to many biocides, disinfectants, and antibiotics [20]. Therefore, inhibiting QS and the virulence factors controlled by it is a primary health objective.

On the other hand, microorganisms have an innate ability to produce reactive oxygen species to promote and maintain their redox cycle and enhance their microbial attachment by forming biofilms. Consequently, oxidative stress is a fundamental driving force for bacteria to transfer from the planktonic (free-living) state to the biofilm layer [21]. Thus, some authors have related the antioxidant property of a sample to its ability to reduce biofilm production by pathogenic bacteria, suggesting the use of antioxidant compounds as an alternative method to treat, prevent, and eradicate biofilms [21,22,23].

Considering these challenges of the food industry, this work aims to determine the chemical compositions of two orange essential oils obtained industrially using different methodologies: the cold-pressed method (EOP) and cold-pressed method followed by steam distillation (EOPD). Moreover, we attempt to determine their potential as antioxidant and antimicrobial agents against planktonic cells and as antipathogenic agents active against biofilm and other virulence factors controlled by QS of *Pseudomonas aeruginosa* and *Staphylococcus aureus*, two significant pathogenic food spoilage bacteria.

## 2. Materials and Methods

### 2.1. Sample

The oranges (*Citrus sinensis* (L.) Osbeck) were cultivated in Entre Rios, Argentina, in 2019, and their Eos (commercial samples) were provided by the Litoral Citrus Company.

Cold-pressed EOs (EOPs) represent 99.9% of the industrial and commercial EOs produced, which are obtained by applying cold pressure to orange peels. The cold-pressing of EOs followed by steam distillation (EOPD) was applied to the liquid discharged from the cold-pressed oil that did not separate in the initial centrifugation process.

### 2.2. Gas Chromatography-Mass Spectrometry

The gas chromatography–mass spectrometry (GC-MS) analysis was carried out with an Agilent 5973N apparatus equipped with a capillary column (95% dimethylpolysiloxane–5% diphenyl), HP-5MS UI (30 m in length and 0.25 mm i.d., with a 0.25 mm film thickness). Here, 2 µL of a mixture containing 20 µL of EO samples in 0.5 mL of dichloromethane (99%, Fisher Scientific, Hampton, NH, USA) was injected. The column temperature program was 60 °C for 5 min, with 3 °C/min increases to 180 °C, then 20 °C/min increases to 280 °C, which was maintained for 10 min. The carrier gas was helium at a flow rate of 1 mL/min. Split mode injection (ratio 1:30) was employed. Mass spectra were taken over the *m*/*z* 30–500 range with an ionizing voltage of 70 eV [24]. The identification of EO components was based on matching their mass spectra peaks with those from the NIST 2005 Mass Spectral Library. The experimental values for Kovats retention indices (RIs), relative to C8–C30 n-alkanes, were determined compared to those from the available literature [25]. They were used as an additional tool to support the MS findings. The percentile presence of components in EO samples was calculated from the peak areas obtained in the area percentage reports (standard processing of chromatograms without replicates), without correction factors, using the normalization method.

### 2.3. Total Phenolic Content

The total phenolic content of the samples was measured spectrophotometrically based on the Folin–Ciocalteu method [22].

### 2.4. Antioxidant Capacity

#### 2.4.1. Phosphomolybdenum Total Antioxidant Activity Assay

The total antioxidant activity of the samples was evaluated using the phosphomolybdenum method, according to Zengin et al. [26]. The sample solution (dil 1/100 DMSO) was combined with 1 mL of reagent solution (0.6 M sulfuric acid, 28 mM sodium phosphate, and 4 mM ammonium molybdate). A control experiment without samples was conducted identically (control). After 90 min of incubation at 95 °C, the absorbance was read at 695 nm. The total antioxidant capacity was calculated from a standard curve (6–50 µg/mL) of ascorbic acid (Biopack, Buenos Aires, Argentina). The results are expressed as ascorbic acid equivalents (AEs).

#### 2.4.2. Nitric Oxide (NO) Scavenging Activity Method

Different concentrations of samples (15–150 µL/mL), sodium nitroprusside (99%, Sigma-Aldrich, St. Louis, MO, USA) (100 mM final concentration), and phosphate buffer (0.2 M, pH 7.4) at a final volume of 300 µL were incubated at 37 °C for 15 min under a light. A control experiment without samples was conducted in an identical manner (control). Then, the reaction mixtures were mixed with Griess reagent, and the absorbance of the chromophore formed was measured at 550 nm after 5 min [22]. The % nitric oxide scavenging activity was calculated using the following equation:

(%) Scavenging = ((DOcontrol − DOsample)/DOcontrol) × 100

where DOcontrol is the absorbance of the mixture reaction containing all reagents except the test compounds and DOsample is the absorbance of the mixture reaction containing the test compounds. The SC_50_ (concentration necessary to scavenge 50% of radical) was calculated using a regression curve (scavenging concentration vs. sample concentration). From an ascorbic acid standard curve (25–200 µg/mL), the ascorbic acid equivalent antioxidant capacity (AEAC) of the samples was calculated.

#### 2.4.3. ABTS Radical Scavenging Method

This assay was performed as described by Re et al. [27] with slight modifications. Samples at different volumes (diluted 1/20 with methanol) were mixed with 200 µL of ABTS radical solution (98%, Sigma-Aldrich, St. Louis, MO, USA) and made up with 96% ethanol to a final volume of 300 µL. A control experiment without samples was conducted identically (control). After a 1 h incubation period at room temperature, the absorbance was recorded at 734 nm using a microplate reader. The percentage of radical scavenging activity was calculated in the following way:

(%) Scavenging = (DOcontrol − DOsample)/DOcontrol) × 100

where DOcontrol is the absorbance of the mixture reaction containing all reagents except the test compounds and DOsample is the absorbance of the mixture reaction containing the test compounds. The SC_50_ was calculated using a regression curve. From a Trolox standard curve (2–8 µg/mL), the Trolox equivalent antioxidant capacity (TEAC) of the samples was calculated.

#### 2.4.4. Cupric-Reducing Antioxidant Capacity (CUPRAC) Method

This assay was determined using the method of Sadeer et al. [28]. The reaction mixture consisted of copper (II) chloride solution (10 mM), neocuproine (98%, Sigma-Aldrich, St. Louis, MO, USA) (7.5 mM), ammonium acetate buffer (1 M, pH = 7), and sample dilutions to reach a final volume of 820 μL. A control experiment without samples was conducted in an identical manner (control). The test tubes were incubated at room temperature (20 to 25 °C) for 30 min. The absorbance at 450 nm was monitored against a blank without neocuproine. The results are expressed as TEAC values using the calibration curve (5–20 µg/mL).

### 2.5. Bacterial Growth Conditions

The strains were obtained from the American Type Culture Collection (ATCC) and Laboratory of Research of Added Value of Regional Products and Foods (LVP) of INBIOFAL (Instituto de Biotecnología Farmacéutica y Alimentaria).

Two *P. aeruginosa* strains were used (ATCC 27853 as a reference and HT5, a multi-antibiotic-resistant strain isolated from a patient with food poisoning). These strains were cultured at 37 °C in Luria–Bertani (LB) medium. Additionally, two *S. aureus* strains were used (ATCC 6538 and HT1 methicillin-resistant). These strains were cultured at 37 °C in Müller–Hinton (MH) medium.

In a microtiter plate, 20 µL of each sample solution (1, 5, 10, 20, and 40 mg/mL) as mixed to arrive at final concentrations of 0.1, 0.5, 1, 2, and 4 mg/mL in wells (*n* = 8) with 180 µL of each strain suspension (OD 0.12 ± 0.01 at 560 nm) from the exponential-phase culture. A vehicle that dissolves EO (DMSO/water, 1:1) was used as the positive control for growth, and the antibiotic ciprofloxacin at low concentration (5 µg/mL) was used as the negative control. The growth was determined at 560 nm (Power Wave XS2, Biotek, Winooski, VT, USA) after 24 h of incubation at 37 °C.

### 2.6. Biofilm Formation Assay

After 24 h of incubation of bacterial cultures prepared as indicated above, the biofilms were stained with 200 μL of an aqueous crystal violet (pa-grade; Cicarelli, Santa Fe, Argentina) solution (0.1%, *w/v*) for 20 min [24,29]. After washing with water, the liquid in the wells was discarded and the material that remained fixed to the polystyrene (biofilm) was washed with distilled water. The crystal violet that adhered to the biofilm in each well was stained using 200 µL of absolute ethanol, and the absorbance was measured at 595 nm using a microtiter plate reader (Multiskan Go, Thermo, Waltham, MA, USA). The biofilm biomass inhibition was calculated relative to the biofilm production in the untreated control culture.

### 2.7. Biofilm Metabolic Activity Assay

Cell viability measured as the bacterial metabolic activity in the biofilm was assessed using the 3-[4,5-dimethylthiazol-2-yl]-2,5-diphenyltetrazolium bromide (MTT) (97.5%, Sigma-Aldrich, St. Louis, MO, USA) reduction assay with some modifications [24,29]. The bacterial biofilm is formed by incubating in all wells 200 µL of each bacterial suspension (OD 560 nm, 0.09 ± 0.02) for 24 h at 37 °C. Subsequently, the bacterial culture is discarded, and the already-formed biofilm remains adhered to the walls. Then, 180 µL of PBS (pH 6.5) and 20 µL of each sample are added to the solution (*n* = 8) per well (final concentrations of 0.1, 0.5, 1, 2, and 4 mg/mL per well), which are then incubated again at 37 °C for 24 h and washed with PBS. To determine bacterial survival, 100 µL of MTT solution (0.5 mg/mL) is added to each well and incubated for 3 or 6 h at 37 °C. If the compound is degraded, the formed purple formazan dissolves in the DMSO and the absorbance is measured at 570 nm. The controls used were the EO vehicle and ciprofloxacin (5 µg/mL).

### 2.8. Elastase Activity and Pyocyanin Quantification

In the cell-free culture, supernatants of each *P. aeruginosa* strain cultivated in the presence and absence of EOs and limonene, pyocyanin, and elastase activities were quantified as described by Díaz et al. [30]. The elastolytic activity in the supernatants was evaluated using the elastin–Congo red conjugate (Sigma–Aldrich, St. Louis, MO, USA) at 495 nm. At the same time, the pyocyanin activity was determined using the chloroform–HCl extraction method and was quantified via absorbance measurements at 520 nm. DMSO-treated cultures were used as controls, and each test was assessed for statistical significance (*n* = 3).

### 2.9. Quantification of N-Acyl Homoserine Lactones (AHL)

Autoinducers are measured for their QS inhibition using the *β*-galactosidase activity assay, using the reporter strain *P. aeruginosa* qsc 119, a mutant donated by P. Greenberg [31], which is incapable of producing its own AHL. This strain responds to exogenous active signal molecules generated by wild-type *P. aeruginosa* strains by producing *β*-galactosidase. Consequently, the activity of *β*-galactosidase is directly related to the concentration of AHL [31]. The AHLs were determined according to a previously reported method [29], using a cell-free culture supernatant obtained from *P. aeruginosa* (ATCC 27853 or HT5) grown individually (*n* = 8) in LB medium in the presence of final concentrations of 4.0, 2.0, 1.0, 0.5, and 0.1 mg/mL of orange EOs and limonene for 24 h. The antibiotic azithromycin was used at a low concentration (5 µg/mL) as a positive control for QS. The *β*-galactosidase activity was measured using the Miller test [32].

### 2.10. Statistical Analysis

Differences in the mean values were evaluated using an analysis of variance (ANOVA). Tukey’s test was used for all pair-wise multiple comparisons of groups. In all analyses, *p* values < 0.05 were considered statistically different (Statistix 7.1, 2002).

## 3. Results

### 3.1. Chemical Composition and Antioxidant Activity

**Figure 1:**
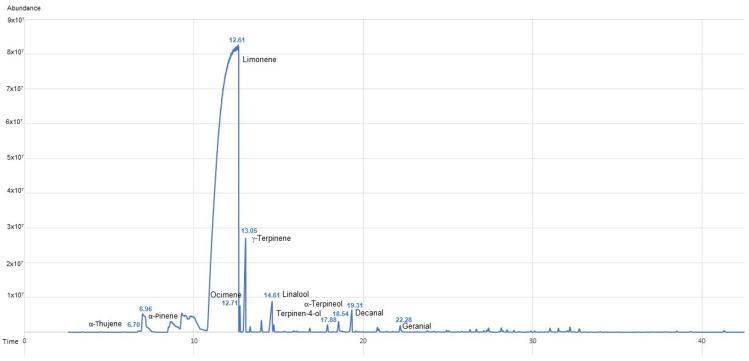
GC chromatogram of orange essential oil obtained using the cold-pressed method followed by steam distillation (EOPD).

**Figure 2:**
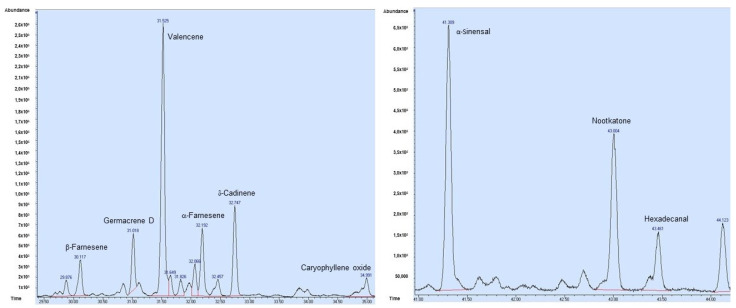
GC chromatogram of the sesquiterpene fractions of orange essential oil obtained using the cold-pressed method (EOP).

In both commercial orange oils (EOP and EOPD), high amounts of monoterpene hydrocarbons were found (96.11 and 97.08%, respectively). The main monoterpene was limonene (90.41 and 89.78%, respectively), followed by myrcene (3.19 and 3.05%, respectively). However, in the EOPD (Figure 1 and Table 1), *γ*-terpinene and *α*-pinene appeared in amounts greater than 1% (1.75 and 1.12%, respectively). Among the oxygenated monoterpenes, the major compound was linalool in both commercial essential oils (0.55 and 0.83%, respectively). In addition, linalyl acetate and sabinene hydrate were only present in respectively EOP, while in respectively EOPD, another compound, mentha-2,8-dien-1-ol, was identified. Small amounts of the sesquiterpenes, both hydrocarbons, and oxygenated sesquiterpenes were found. Minimum qualitative and quantitative differences in this phytochemical group were observed in respectively EOP and EOPD (0.55 vs. 0.49%, 0.12 vs. 0.34%, and 0.12 vs. 0.03%, respectively). Concerning the sesquiterpene hydrocarbons, *δ*-elemene and *γ*-muurolene were only found in the EOPD, whereas germacrene D, caryophyllene oxide, and nootkatone were detected among the sesquiterpenes and oxygenated sesquiterpenes in the EOP (Figure 2 and Table 1). In addition, the sesquiterpene hydrocarbon valencene showed a three-fold higher concentration in EOP than EOPD (Table 1). Other constituents such as hexadecanal, hexadecanoic acid, and tetracosane were only found in EOP.

**Figure 3:**
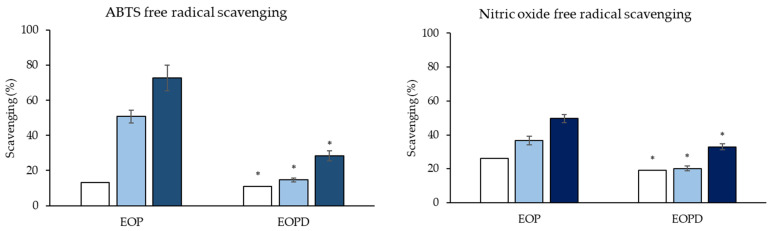
Free radical scavenging activity levels of different essential oil concentrations. EOP: Essential oil obtained by cold-pressed method; EOPD: essential oil obtained by cold-pressed method followed by steam distillation. Concentrations assayed for ABTS in EOP at 1.5 (□), 8 (■), and 12 (■) µL/mL and EOPD at 12 (□), 15 (■), and 30 (■) µL/mL. Concentrations assayed for nitric oxide in EOP at 15 (□), 30 (■), and 60 (■) µL/mL and EOPD at 30 (□), 60 (■), and 150 (■) µL/mL. Data are presented as means ± SEMs (*n* = 3); * represents significant differences between the oils, according to Tukey’s test (*p* < 0.05).

Different assays were carried out to evaluate the antioxidant properties of the EOP and EOPD. The ABTS assay based on the scavenging of a stable free radical (ABTS•+), CUPRAC assay based on the capacity of antioxidants to reduce ions copper, nitrite assay based on the scavenging of free radicals focused on nitrogen (•NO), and phosphomolybdenum assay based on the reduction of Mo(VI) to Mo(V). *C. sinensis* oils showed significant antioxidant potential in various experimental models by scavenging free radical and nitrogen species and reducing metals. The antioxidant results and phenolic content (Table 2 and Figure 3) showed that although both oils presented similar reducing capacity levels, the radical scavenging ability was higher for the EOP (SC_50_ 8 and 65.5 µL/mL for ABTS and NO, respectively) than EOPD. This could be related to their chemical compositions, since although both have similar terpene contents, the total polyphenol concentration of EOP is eight times higher than for EOPD. In addition, EOP contains greater amounts of oxygenated compounds, mainly the antioxidants nootkatone (not found in EOPD) and valencene (0.169 vs. 0.057).

### 3.2. Planktonic Growth and Total Biofilm Formation by S. aureus and P. aeruginosa in the Presence of Orange Oils

**Figure 4:**
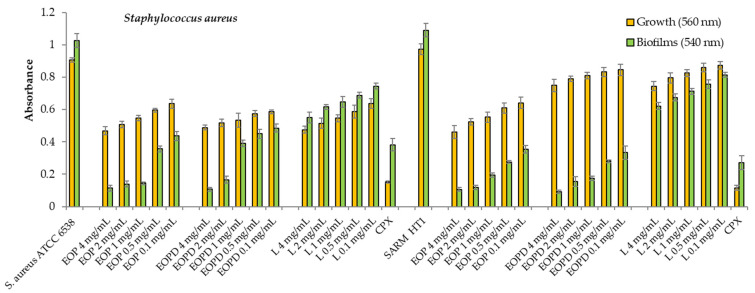
Growth and biofilms of *Staphylococcus aureus* (ATCC 6538 and methicillin-resistant HT1 strains). EOP: Essential oil obtained by cold-pressed method; EOPD: essential oil obtained by cold-pressed method followed by steam distillation; L: Limonene; CPX: ciprofloxacin. Data are presented as means ± SEMs (*n* = 8) of three independent experiments. All experiments showed significant differences compared to respective controls (*p* < 0.05).

The EOP, EOPD, and their main component, limonene, moderately inhibited the planktonic growth of both *S. aureus* strains (Figure 4). However, the natural orange products inhibited bacterial biofilm formation by more than 50% for both bacteria at all concentrations assayed. The EOP’s inhibition values ranged from 89 to 57% for the concentration range of 4 to 0.1 mg/mL, while the EOPD’s inhibition values ranged from 90 to 53%. A dose-dependent effect until 2 mg/mL was observed for both strains. Limonene, for its part, had a lower inhibitory effect range of 46 to 25% at the same range of concentrations (Figure 4).

**Figure 5:**
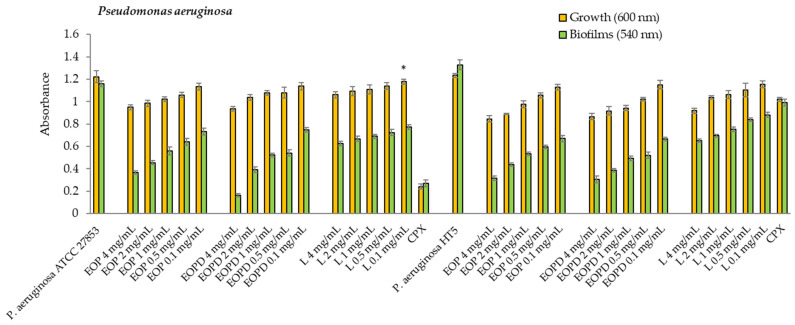
Growth and biofilms of *Pseudomonas aeruginosa* (ATCC 27853 and HT5 strains). EOP: Essential oil obtained by cold-pressed method; EOPD: essential oil obtained by cold-pressed method followed by steam distillation; L: limonene; CPX: ciprofloxacin. Data are presented as means ± SEMs (*n* = 8) of three independent experiments. All experiments show significant differences compared to the respective controls (*p* < 0.05), except the sample with asterisk (*).

The orange EOs reduced the growth of the *P. aeruginosa* strains from 31 to 6% in a concentration range of 4 to 0.1 mg/mL. In comparison, limonene caused decreases of 26 to 4% (Figure 5). However, all samples significantly inhibited the development of the *P. aeruginosa* ATCC 27853 biofilms. The EOP produced inhibition effects of 69 to 37%, the EOPD produced inhibition effects of 87 to 36%, and limonene showed the lowest inhibitory effects (46–33%) at the tested concentrations. Concerning the HT5 strain, the EOP and EOPD produced 77 to 50% inhibition rates for the concentration range of 4 to 0.1 mg/mL. On the other hand, the limonene caused inhibition rates of 51 to 25% in the same range of concentrations (Figure 5). The biofilm formation decreases were dose-dependent for both strains.

No significant (*p* > 0.05) differences were observed between the EOs (EOP and EOPD) based on the planktonic growth and biofilm biomass formation of the different bacterial species studied, except for the biofilm formation of the *P. aeruginosa* ATCC 27853 strain in the presence of 4 mg/mL of EO.

### 3.3. S. aureus and P. aeruginosa Biofilm Metabolic Activity in the Presence of Orange Oils

The tested products moderately inhibited the metabolic activity of *S. aureus* in the phenotype biofilm (Table 3). The EOP, EOPD, and limonene diminished the viability rates of the ATCC 6538 strain in the concentration range of 4 to 0.1 mg/mL by 42 to 27%, 48 to 30%, and 38 to 17%, respectively. The effects on the methicillin-resistant strain were 37–21%, 41–23%, and 42–26% for the EOP, EOPD, and limonene, respectively, at the tested concentrations. Likewise, the EOP, EOPD, and the main constituent of both, limonene, significantly inhibited the metabolic activity of *P. aeruginosa* in the phenotype biofilm (Table 3). At 4 mg/mL, the EOP, EOPD, and limonene inhibited the cell viability rates by 68, 65, and 36% for the ATCC 27853 strain; and by 57, 56, and 41% for the strain HT5, respectively. These results show a dose-dependent effect for all strains.

The EOP and EOPD exerted a similar (*p* > 0.05) action on the cell metabolic activity in a preformed biofilm of the selected strains.

### 3.4. Elastase, Pyocyanin, and Autoinducer Production by P. aeruginosa in the Presence of Orange Oils

**Figure 6:**
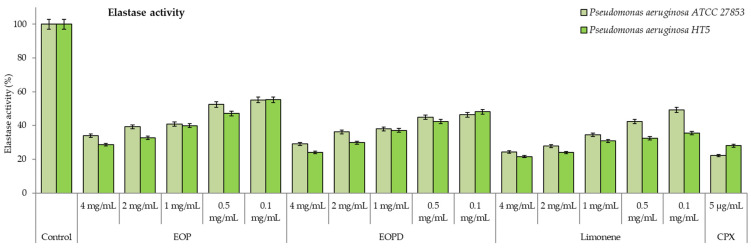
Elastase activity of *Pseudomonas aeruginosa* ATCC 27853 and HT5 strains. EOP: Essential oil obtained by cold-pressed method; EOPD: essential oil obtained by cold-pressed method followed by steam distillation; L: limonene; CPX: ciprofloxacin. Data are presented as means ± SEMs (*n* = 8) of three independent experiments. All experiments show significant differences compared to respective controls (*p* < 0.05).

The orange essential oils and limonene significantly inhibited the enzyme elastase, a key virulence factor of *P. aeruginosa* in the spread of infection (Figure 6). For both strains, the effect was significant and greater than 45%, even at the lowest concentration tested (0.1 mg/mL), reaching an elastolytic activity level close to 70% at 4 mg/mL for all natural orange products. No statistical differences were observed for elastase activity between the essential oils (*p* > 0.05). Likewise, the dose-dependent effect on the elastase production was only notable for the HT5 strain.

Concerning the QS-dependent pyocyanin production of *P aeruginosa*, the EOP, EOPD, and limonene significantly inhibited the production of this toxic pigment. For both strains at the maximum concentration, the effects of EOP and EOPD equaled 65%, while for limonene the rate was 57%. At the lowest concentration, the effects were still significant, at close to 40% for both oils and less than 30% for limonene (Table 4). The results show a clear dose-dependent decrease in this virulence factor. No significant variations were observed in the production of pyocyanin between the essential oils (*p* > 0.05).

**Figure 7:**
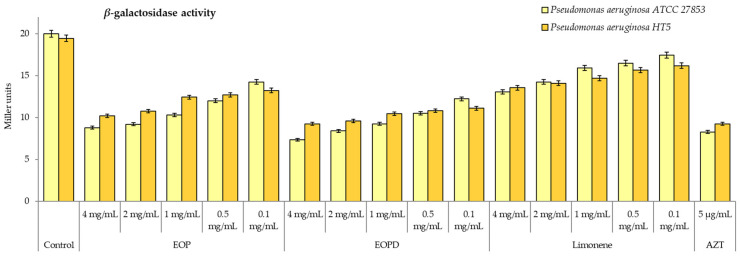
The *β*-galactosidase activity of *Pseudomonas aeruginosa* ATCC 27853 and HT5 strains. EOP: Essential oil obtained by cold-pressed method; EOPD: essential oil obtained by cold-pressed method followed by steam distillation; L: limonene; AZT: azithromycin. Data are presented as means ± SEMs (*n* = 8) of three independent experiments. All experiments show significant differences compared to respective controls (*p* < 0.05).

As seen in Figure 7, both sweet orange EOs and limonene can reduce the *β*-galactosidase activity of *P. aeruginosa*, which depends on the QS mechanism. This fact indicates that EOP, EOPD, and limonene can inhibit the production of AHLs in both strains and can interrupt the bacterial communication. In *P. aeruginosa* ATCC 27853, inhibition rates of 56 to 29% and 63 to 39% by EOP and EOPD, respectively, were observed in the concentration range of 4 to 0.1 mg/mL. Meanwhile, the principal constituent of the orange essential oils, limonene, had a lower inhibitory effect range of 35 to 13% for the same range of concentrations. For the multiresistant *P. aeruginosa* HT5, the EOP and EOPD caused 47 to 32% and 52 to 43% inhibition effects, respectively, for the 4 to 0.1 mg/mL concentration range. The effects of both EOs on the AHL production were dose-dependent; however, at the low concentrations, the EOPD showed greater inhitibion of both strains (*p* < 0.05). In comparison, limonene reduced the production of autoinducers from 30 to 17% in the same range of concentrations.

## 4. Discussion

The chemical compositions of *C. sinensis* EOs vary depending on the extraction method used. Nevertheless, they also depend on the region where the sample was taken and the cultivar was analysed. The composition results of the volatilizable fraction of orange EOs agreed with previous studies in which limonene was the main component, with an average concentration range of 75 to 97.3% [33,34,35]. In concordance with our results, myrcene (3.2–3.1%) was the second most abundant monoterpene hydrocarbon in *C. sinensis* EOs from Argentina, the United States, and Nepal [34,35]. Additionally, the monoterpenes *α*-pinene and *γ*-terpinene were observed in appreciable amounts. However, in *C. sinensis* EOs from Tunisia, *β*-pinene (1.45–1.82%) was the second most abundant monoterpene hydrocarbon, followed by *α*-pinene and sabinene [33]. The second main group contained the oxygenated monoterpenes with linalool as the main compound [34,35].

The cold-pressing technique is a unique process applied to extract edible oils from various oily seeds, kernels, peels, and fruits. This technique is important in producing specialty oils with typical characteristic aroma compounds and functional and nutritional compositions. Cold-pressed oils are preferred for their desirable flavour (*α*- and *β*-sinensal) characteristics, with their antioxidant components (phenolic compounds, nootkatone, valencene) having therapeutic effects [11,36]. Indeed, the cold-pressing method is common and one of the best techniques for isolating EOs. Using this method, pressure is applied to the plant sample without enhancing the temperature. This extraction technique has significant advantages compared to other extraction techniques, such as the lower energy costs and the fact it does not require a solvent or advanced equipment. The extraction methods influence the EO composition; as reported by González-Mas et al. [37], steam distillation could result in the loss of certain compounds with high molecular weights and low volatility, such as certain phenolic compounds. This observation is consistent with our results, where the concentration of phenolic compounds was eight times higher in the EOP than in the EOPD.

In addition, several studies have reported a relationship between *Citrus* EOs’ compositions and their antioxidant properties [38]. Some authors assigned the antioxidant activity of *Citrus* EOs to the volatilizable fractions, particularly monoterpenes, while other researchers attributed it to the presence of phenolic compounds [13,39]. Based on the results obtained in this work with *C. sinensis* oils, we suggest that the free radical scavenging activity is conferred to antioxidant compounds such as polyphenols and valencene (present in higher concentrations in EOP) and nootkatone (only present in EOP). Meanwhile, their reducing capacity is attributed to the terpenes present in similar proportions in both oils. Frasinetti et al. [40] attributed the antioxidant capacity of the bitter orange, sweet orange, lemon, and mandarin EOs to the presence of monoterpenes, the main compounds found in these oils. Moreover, some reports have shown that monoterpenes, such as limonene, *α*- and *γ*-terpinene, terpinolene, geraniol, *β*-pinene, myrcene, *α*-terpineol, and linalool, were mainly responsible for the antioxidant potential of many plant oils, including *Citrus* oils [41,42,43,44]. In addition, oxygenated monoterpenes with different functional groups, such as phenols, alcohols, aldehydes, ethers, esters, and ketones, contribute significantly to the antioxidant properties of the *Citrus* EOs [41]. Raspo et al. [34] explained that these minor components or the synergy between them might be the cause of the antioxidant potential of *Citrus* EOs.

On the other hand, Noshad et al. [45] stated that phenolic compounds act as electron donors in free radical reactions and are often correlated with the antioxidant effects of EOs. In addition, Bonilla and Sobral [46] reported that the phenolic components of cinnamon EOs are capable of quenching reactive oxygen species to delay lipid oxidation. Moreover, the activities of monoterpene phenols have been associated with their phenolic structures and redox properties, which perform a fundamental function in the decomposition of peroxides, in addition to free radical neutralization [47].

In agreement with the present results, Raspo et al. [34] found that Argentinian orange EOs extracted by hydrodistillation showed antioxidant capacity. The authors used ABTS (16 mg Trolox equivalent/mL), DPPH (8 mg Trolox equivalent/mL), CUPRAC (3.5 mg Trolox equivalent/mL), and FRAP assays (0.15 mmol AA/mL). To our knowledge, there are no studies on the purifying capacity of nitric oxide by *C. sinensis* EOs.

The antimicrobial activity levels of the commercial orange oils obtained by cold-pressing (EOP) and cold-pressing followed by a hot distillation system (EOPD) were moderate and higher against *S. aureus* than *P. aeruginosa*. In concordance with our results, the *C. sinensis* EOs were more antimicrobial against pathogenic and food spoilage Gram-positive bacteria than Gram-negative bacteria [48]. It is well known that terpenoid and phenolic compounds have a wide range of biological activities, including antibacterial and antimicrobial activities. In particular, the pure main compounds limonene and myrcene from our sample have been reported as antibacterial agents against Gram-positive strains [49,50].

In addition, different *Citrus* EOs showed better antimicrobial effects against Gram-positive than Gram-negative bacteria [51]. Although limonene is the main constituent, the antimicrobial activity of orange essential oils was higher. This could be attributed mainly to the terpenes, and more specifically to a synergism between their components. Therefore, the antibacterial effects of these EOs are not uniform against different bacteria because they depend on the chemical composition; that is, the antimicrobial activity of the EOs depends on the presence of specific phytochemical components and their interactions, and these components vary according to the maturation stage of the plant. For example, when obtained from ripe fruit, the sweet orange essential oil is more effective against *P. aeruginosa* [52]. In another study, the behaviours of the compounds present in the essential oil of *C. sinensis* were compared according to the plant’s maturation stage. It was observed that the limonene values did not vary, but the minority compounds were affected. Therefore, an essential oil’s inhibitory activity results from a complex interaction between its different components, which can produce additive, synergistic, or antagonistic effects [53]. *Citrus* EOs such as grapefruit, bergamot, orange, lime, and lemon inhibit the growth of common foodborne and medically important bacterial pathogens, mostly having high minimal inhibitory concentration (MIC) values [51,54,55]. On the other hand, neither limonene nor *Citrus* EOs are bactericidal, even at high concentrations [56].

However, the orange EOs could be considered antipathogenic because they significantly inhibit biofilm production, virulence factors, and QS signals (AHLs). These results suggest that the inhibition effects displayed by EOP and EOPD did not relate exclusively to their action on the growth and that the QS mechanism was involved, coherently with previous studies on plant natural products [57,58]. Compounds with antipathogenic capacities instead of being involved in killing bacteria or stopping their growth act by controlling bacterial virulence factors such as the biofilm and elastase activity and prevent the development of resistant strains [59].

With respect to the biofilm formation, both essential oils interfered with its development in the same manner. The EOPD showed 18% greater inhibition of the *P. aeruginosa* ATCC biofilm than the EOP at 4 mg/mL (*p* < 0.05). It is important to note that the EOPD had a higher total content of monoterpenes and oxygenated monoterpenes (Table 1). Specifically, in relation to the linalool contents (0.55 vs. 0.83% in EOP and EOPD, respectively) and other compounds such as terpinen-4-ol (0.03 vs. 0.11), *α*-terpinol (0.08 vs. 0.20) and mentha-2,8-dien-1-ol were only found in EOPD. Concerning the minor sesquiterpene hydrocabons, *δ*-elemene and *γ*-muurolene were only found in EOPD.

It is important to note that the main compounds limonene (found in both EOs) and linalool have relevant antibiofilm and anti-QS properties against *P. aeruginosa* [29,59,60], which could explain the potential synergistic effect between them and the differential behaviours that were observed.

In agreement with the present results, previous studies have shown that *Citrus* EOs can be effective against bacterial biofilms. *Citrus limon* oils inhibited specific biofilm production and bacterial metabolic activities into biofilm in a dose-dependent manner for *P. aeruginosa* strains. Moreover, these EOs diminished 50% of the elastase activity at 0.1 mg/mL and decreased the pyocyanin biosynthesis. Additionally, another virulence factor, the swarming motility, was completely inhibited by 2 mg/mL. The results were correlated with the observed decrease (29–55%, 0.1–4 mg/mL) in QS signal synthesis [29]. *Citrus paradisi* (grapefruit) essential oils at low concentrations (0.1 mg/mL), mainly obtained by cold-pressing (EOP), were able to inhibit the biofilm establishment and the bacterial survival in the biofilm previously formed by *P. aeruginosa*. These EOs also reduced the *P. aeruginosa’s* AHL production and elastase activity [59]. In addition, grapefruit EO has been shown to inhibit the formation of enterohemorrhagic *Escherichia coli* biofilms, while lemon EO inhibited both monomicrobial and mixed biofilms formed by *E. coli* and *Bacillus cereus* [61,62]. In the case of the mandarin EOs, they were not able to inhibit the *P. aeruginosa* growth at 4 mg/mL. However, they significantly inhibited the *P. aeruginosa* biofilm formation at 0.1 mg/mL, as well as the biofilm cell viability (41%), AHL production (33%), and elastase enzyme activity (75%) [24]. It was reported that many EOs could affect bacterial virulence through interference with QS. Rose, geranium, lavender, and rosemary EOs potently inhibited QS; eucalyptus and *Citrus* EOs moderately reduced violacein production; and the chamomile, orange, and juniper oils were ineffective [63]. Pekmezovic et al. [56] demonstrated that the QS inhibition in *P. aeruginosa* occurred through interference with AHL pathways. Several EOs have shown their ability to interfere with bacterial QS signalling and inhibit biofilm formation [61,62,64,65]. As QS inhibitors do not kill or inhibit bacterial growth, these agents have an advantage because they do not impose a selective pressure for resistance development compared to antibiotics [66]. Therefore, the fact that *C. sinensis* EOs have anti-QS activity is very significant, since it would allow the elimination of pathogens with resistance mechanisms. This is very important considering that they could be used in the food industry since they are safe, and many foods tolerate the presence of *Citrus* essential oils. Moreover, many of them are applied in industrial fields in various products, including cosmetics, drugs, foods, and beverages, due to their broad spectrum of biological activities, such as their antibacterial and antifungal activities [33].

Guo et al. [38] did not find a clear relationship when evaluating and comparing the chemical compositions and antimicrobial and antioxidant activities of essential oils from fourteen species of *Citrus*. Despite their promising antioxidant activities, the samples showed only marginal antimicrobial properties. In concordance with what was found in this work, both oils have poor antibacterial activity and significant antipathogenic and antioxidant capacity.

It is crucial to note that the free radical scavengers and antioxidants could also be linked to the promising attenuation of the quorum detection mechanism, because QS is activated by stress factors such as free radicals and oxidative agents.

## 5. Conclusions

The EOP and EOPD showed antioxidant activity by reducing metals, and particularly the EOP by also neutralizing free radicals. On the other hand, they partially affected the bacterial growth while strongly inhibiting the biofilm formation and viability of sessile bacteria living in a pre-existent biofilm (Gram-negative and Gram-positive). Moreover, the inhibition of AHL formation is reflected in the control of the production of other virulence factors such as elastase and pyocyanin. Therefore, they could represent natural and safe alternatives to extend the shelf life of food products by preventing oxidation and contamination by pathogens that spoil food, meaning the sweet orange EOs can be considered as an innovative dual strategy for food preservation.

## Figures and Tables

**Table 1:** Chemical constituents of essential oils obtained from fruit peels of *Citrus sinensis* (L.) Osbeck (Rutaceae).

Compounds	RI ^a^	KI ^b^	Identification Methods	Relative Content (%)	
				EOP	EOPD
**Monoterpene hydrocarbons**				**96.111**	**97.080**
*α*-thujene	926	930	RI, MS	0.017	0.060
*α*-pinene	933	939	RI, MS	0.952	1.119
Sabinene	972	975	RI, MS	0.140	0.076
*β*-pinene	976	979	RI, MS	0.500	0.935
Myrcene	989	990	RI, MS	3.188	3.049
Limonene	1041	1039	RI, MS	90.408	89.781
Ocimene	1063	1050	RI, MS	0.061	0.153
*γ*-terpinene	1069	1059	RI, MS	0.838	1.752
Terpinolene	1088	1088	RI, MS	0.007	0.155
**Oxygenated monoterpenes**				**1.238**	**1.457**
Sabinene hydrate	1074	1070	RI, MS	0.006	-
Linalool	1101	1095	RI, MS	0.551	0.831
Mentha-2,8-dien-1-ol	1121	1122	RI, MS	-	0.020
*cis*-limonene oxide	1134	1136	RI, MS	0.011	0.017
*trans*-limonene oxide	1138	1142	RI, MS	0.029	0.012
Citronellal	1153	1153	RI, MS	0.078	0.050
Terpinen-4-ol	1176	1177	RI, MS	0.034	0.109
*α*-terpinol	1188	1188	RI, MS	0.077	0.200
Neral	1239	1238	RI, MS	0.125	0.064
Carvone	1241	1243	RI, MS	0.017	0.052
Linalyl acetate	1254	1257	RI, MS	0.001	-
Geranial	1269	1267	RI, MS	0.221	0.011
*α*-terpinyl acetate	1345	1349	RI, MS	0.011	0.007
Citronellyl acetate	1350	1352	RI, MS	0.005	0.012
Neryl acetate	1361	1361	RI, MS	0.027	0.032
Geranyl acetate	1380	1381	RI, MS	0.019	0.023
Limonen-10-yl-acetate	1405	1395	RI, MS	0.026	0.017
**Sesquiterpene hydrocarbons**				**0.552**	**0.487**
*δ*-elemene	1333	1338	RI, MS	-	0.016
*α*-copaene	1370	1376	RI, MS	0.037	0.042
*β*-cubebene	1384	1388	RI, MS	0.035	0.025
*β*-elemene	1385	1390	RI, MS	0.025	0.052
*β*-caryophyllene	1411	1419	RI, MS	0.043	0.034
*β*-copaene	1422	1432	RI, MS	0.054	0.024
*α*-bergamotene	1430	1434	RI, MS	0.011	0.008
*α*-humulene	1446	1454	RI, MS	0.009	0.016
*β*-farnesene	1452	1456	RI, MS	0.023	0.016
Germacrene D	1474	1480	RI, MS	0.034	-
*γ*-muurolene	1474	1479	RI, MS	-	0.057
Valencene	1486	1496	RI, MS	0.169	0.057
Bicyclogermacrene	1489	1500	RI, MS	0.012	0.011
*α*-muurolene	1492	1500	RI, MS	0.009	0.007
*α*-farnesene	1502	1505	RI, MS	0.034	0.069
*δ*-cadinene	1516	1523	RI, MS	0.055	0.049
Germacrene B	1549	1561	RI, MS	0.002	0.004
**Oxygenated sesquiterpenes**				**0.122**	**0.034**
Caryophyllene oxide	1574	1583	RI, MS	0.008	-
*β*-sinensal	1690	1699	RI, MS	0.053	0.011
*α*-sinensal	1746	1756	RI, MS	0.038	0.023
Nootkatone	1794	1806	RI, MS	0.023	-
**Other compounds**				**0.818**	**0.716**
Octanol	1076	1068	RI, MS	0.019	0.062
Nonanal	1104	1100	RI, MS	0.100	0.088
Decanal	1203	1201	RI, MS	0.517	0.431
Octanol acetate	1210	1213	RI, MS	0.016	0.014
2*E*-decenal	1259	1263	RI, MS	0.009	0.012
Undecanal	1302	1306	RI, MS	0.024	0.018
2*E*,4*E*-decadienal	1311	1315	RI, MS	0.005	0.009
Methyl-*N*-methyl anthranilate	1399	1406	RI, MS	-	0.009
Dodecanal	1403	1408	RI, MS	0.093	0.059
2*E*-dodecenal	1461	1466	RI, MS	-	0.006
Hexadecanal	1808	1817	RI, MS	0.009	-
Hexadecanoid acid	1961	1960	RI, MS	0.019	-
Tricosane	2283	2300	RI, MS	0.005	0.008
Tetracosane	2385	2400	RI, MS	0.002	-
**Total VOCs**				**98.841**	**99.774**

^a^ RI: Retention index relative to C8–C30 n-alkane on HP-5MS column; ^b^ KI: Kovats retention index; VOCs: volatilizable organic compounds; EOP: essential oil obtained by cold-pressed method; EOPD: essential oil obtained by cold-pressed followed by steam distillation.

**Table 2:** Antioxidant activity of *Citrus sinensis* oils by total phenolic composition.

Oils	Phenolic Compounds	Reducing Capacity		Scavenging Capacity	
		Phosphomolybdenum Assay	CUPRAC Assay	Nitric Oxide Radical	ABTS Radical
	µg GAE/mL EO	mg AE/mL EO	mg TEAC/mL EO	mg AEAC/mL EO	mg TEAC/mL EO
EOP	84.80 ± 7.20	245.11 ± 18.60	0.55 ± 0.02	2.10 ± 0.45	0.55 ± 0.10
EOPD	10.53 ± 1.20 *	257.12 ± 10.60	0.52 ± 0.006	0.35 ± 0.08 *	0.07 ± 0.004 *

GAE: Gallic acid equivalent; AE ascorbic acid equivalent; AEAC: ascorbic acid equivalent antioxidant capacity; TEAC: Trolox equivalent antioxidant capacity. Results are expressed as means ± standard deviations (*n* = 3). EOP: Essential oil obtained by cold-pressed method; EOPD: essential oil obtained by cold-pressed method followed by steam distillation. Data are presented as means ± SEMs (*n* = 3); * indicates significant differences between samples, according to Tukey’s test (*p* < 0.05).

**Table 3:** Biofilm metabolic activity (%) rates of *Staphylococcus aureus* and *Pseudomonas aeruginosa* strains.

Samples	*Staphylococcus aureus*		*Pseudomonas aeruginosa*	
	ATCC 6538	HT1	ATCC 27853	HT5
**Control**	100 ± 2.07	100 ± 1.97	100 ± 1.57	100 ± 2.00
**EOP**				
4 mg/mL	58.93 ± 1.19	63.09 ± 5.19	31.56 ± 7.00	43.21 ± 4.96
2 mg/mL	59.57 ± 1.97	70.14 ± 1.96	43.52 ± 3.88	49.23 ± 6.65
1 mg/mL	64.06 ± 3.30	73.22 ± 5.57	57.40 ± 3.62	58.87 ± 3.27
0.5 mg/mL	69.71 ± 3.00	76.59 ± 1.00	67.97 ± 2.78	61.62 ± 6.05
0.1 mg/mL	72.78 ± 1.88	78.97 ± 1.69	79.45 ± 2.35	66.41 ± 4.79
**EOPD**				
4 mg/mL	49.84 ± 2.25	59.06 ± 1.95	34.49 ± 7.04	43.96 ± 7.55
2 mg/mL	59.28 ± 1.35	63.32 ± 6.28	49.25 ± 2.64	50.55 ± 3.03
1 mg/mL	63.83 ± 2.07	69.57 ± 2.88	56.64 ± 3.80	57.92 ± 2.67
0.5 mg/mL	65.76 ± 5.25	73.22 ± 1.21	72.93 ± 2.19	61.59 ± 4.90
0.1 mg/mL	70.67 ± 1.57	76.57 ± 2.16	80.67 ± 1.58	71.80 ± 1.91
**Limonene**				
4 mg/mL	61.78 ± 3.57	58.29 ± 1.82	64.20 ± 3.73	58.56 ± 3.14
2 mg/mL	66.13 ± 3.36	60.70 ± 2.62	68.45 ± 2.66	66.50 ± 2.61
1 mg/mL	72.41 ± 1.94	63.14 ± 1.68	72.13 ± 1.70	71.30 ± 2.00
0.5 mg/mL	74.73 ± 2.16	70.61 ± 4.31	77.54 ± 1.52	78.99 ± 3.53
0.1 mg/mL	83.55 ± 2.14	74.47 ± 4.37	88.23 ± 1.59	84.74 ± 1.70
Ciprofloxacin 5 µg/mL	55.08 ± 2.81	55.16 ± 2.73	19.82 ± 3.12	64.22 ± 0.80

EOP: Essential oil obtained by cold-pressed method; EOPD: essential oil obtained by cold-pressed method followed by steam distillation. Data are presented as means ± SEMs (*n* = 8) of three independent experiments. All experiments show significant differences compared to respective controls (*p* < 0.05).

**Table 4:** Inhibition (%) of the production of the *Pseudomonas aeruginosa* virulence factor pyocyanin.

Sample	*P. aeruginosa* ATCC 27853	*P. aeruginosa* HT5
EOP 4 mg/mL	62.30 ± 2.05	68.02 ± 1.03
EOP 0.1 mg/mL	37.12 ± 3.00	40.07 ± 2.14
EOPD 4 mg/mL	65.21 ± 1.03	69.19 ± 0.00
EOPD 0.1 mg mL	41.42 ± 2.11	43.11 ± 4.08
Limonene 4 mg/mL	57.45 ± 0.05	58.23 ± 3.01
Limonene 0.1 mg/mL	30.00 ± 1.10	23.44 ± 3.23

EOP: Essential oil obtained by cold-pressed method; EOPD: essential oil obtained by cold-pressed method followed by steam distillation. Data are presented as means ± SEMs (*n* = 3) of three independent experiments. All experiments show significant differences compared to respective controls (*p* < 0.05).

## Data Availability

The data are contained within the article.
